# Newborn and infant hearing screening for early detection of hearing loss in Nairobi, Kenya

**DOI:** 10.4314/ahs.v24i1.28

**Published:** 2024-03

**Authors:** Serah Ndegwa, Debara Tucci, James Lemons, Florence Murila, Susan Shepherd, Moses Mwangi, Isaac Macharia, John Ayugi

**Affiliations:** 1 Department of Surgery, University of Nairobi, Kenya; 2 National Institute on Deafness and Other Communication Disorders (NIDCD); 3 Indiana University School of Medicine; 4 Department of Paediatrics, University of Nairobi, Kenya; 5 Indiana University and Purdue University at Indianapolis; 6 Kenya Medical Research Institute

**Keywords:** hearing screening, newborn, infant, hearing loss, early detection, Kenya

## Abstract

**Background:**

Early detection of hearing loss and subsequent intervention leads to better speech, language and educational outcomes giving way to improved social economic prospects in adult life. This can be achieved through establishing newborn and infant hearing screening programs.

**Objective:**

To determine the prevalence of hearing loss in newborns and infants in Nairobi, Kenya.

**Methods:**

A cross-sectional pilot study was conducted at the National hospital and at a sub county hospital immunization clinic. A total of 9,963 babies aged 0-3 years, were enrolled in the hearing screening program through convenient sampling over a period of nine months. A case history was administered followed by Distortion Product Oto-acoustic emissions (DPOAEs) and automated auditory brainstem response (AABR) hearing screening.

**Results:**

The screening coverage rate was 98.6% (9963/10,104). The referral rate for the initial screen was 3.6% (356/ 9,963), the return rate for follow-up rescreening was 72% (258 babies out of 356) with a lost to follow-up rate of 28% (98/356). The referral rate of the second screen was 10% (26/258). All the 26 babies referred from the second screen returned for diagnostic hearing evaluation and were confirmed with hearing loss, yielding a prevalence of 3/1000.

**Conclusions:**

Establishing universal newborn and infant hearing screening programs is essential for early detection and intervention for hearing loss. Data management and efficient follow-up systems are an integral part of achieving diagnostic confirmation of hearing loss and early intervention.

## Introduction

Newborn and infant hearing screening for the early detection of permanent hearing loss is considered an important component of early childhood healthcare in developed countries 1,2. If undetected early, hearing loss in children is known to have adverse effects on the development of speech, language, cognitive and psychosocial skills, with a subsequent negative impact on educational achievements and future employment prospects 3-6. The World Health Organization (W.H.O) estimates that at least 466 million people globally have disabling hearing loss with 34 million of these being children 7. Hearing loss is now rated as the fourth leading cause of years lived with disability, having risen up from 11^th^ leading cause in 2010 7,8. Approximately 90% of people with disabling hearing loss defined as hearing loss in the better ear of >30 dBHL in children (0-15 years) and >40dBHL in adults, live in low or middle-income countries 7,9. In Sub-Saharan Africa, the prevalence of disabling hearing loss is estimated at 4.55 % among all ages 7. The W.H.O classifies hearing loss as Mild (26-40 dBHL), Moderate (41-60 dBHL), Severe (61-80dBHL) and Profound (≥81dBHL) 9. Hearing loss is referred to as a hidden disability due to its invisible nature which often causes it go undetected and untreated. In developing countries, the reported prevalence ofpermanent hearing loss in newborn babies ranges between 3-6/1000 while in developed countries the range is between 1-3/1000 11-15. About 50% of all hearing loss is preventable through preventive strategies such as immunization, health education and improving maternal and child health services 16,17.

Undiagnosed hearing loss of any severity or unilateral, can lead to delays in speech and language development in children 3,18. The goal of newborn and infant hearing screening is to enable early identification and intervention for hearing lss in newborn and infants within the critical time of language development.^9^ Hearing screening programs in low- and middle-income countries are lacking and detection of hearing loss in children is mainly through parental suspicion 20,21. The Joint Committee of Infant Hearing (JCIH) recommends screening by one month, a comprehensive audiological evaluation by three months and appropriate intervention by six months of age 22,23. Hearing screening for newborns is done in the maternity unit prior to hospital discharge and can also be done at immunization clinics 24,25. Otoacoustic Emissions (OAEs) and Automated Auditory Brainstem Response (AABR) are the two objective physiological tests of auditory function that are widely used in universal newborn hearing screening programs 23,26. They are reliable, cost-effective, non-invasive, simple to administer and can be effectively conducted by primary healthcare workers who have undergone some training. 26,27. There are four categories of screening protocols available for use at the first stage of UNHS programs: (a)OAEs only, (b)AABR only, (c) OAE followed by AABR when OAE refers in one or both ears (d) both OAE and AABR, where a pass is required for both the OAE and AABR screening in one or both ears 28. The OAE screening takes about 3-5minutes, is less expensive and has higher referral rates in comparison to AABR screening which takes about 12-14 minutes, is more expensive but has lower referral rates 29,30. AABR screening is recommended for babies admitted to NICU for > 5 days as prolonged admission to NICU is associated with auditory neuropathy 22. Identification of hearing loss is dependent on getting good return rates for infants referred for diagnostic hearing evaluation. The JCIH recommends that The JCIH recommends that a Universal hearing screening program should have a coverage of at least 95%, a follow-up return rate of at least 70% and a referral rate for diagnostic audiological evaluation not exceeding 4% 22,23. Efficient tracking systems and good communication between the health professionals and parents have been found to be necessary in ensuring high follow-up return rates 16, 28.

Kenya is classified as a lower middle-income country with a population of 47.5 million and 1.4 million births per year 31 In 2018, the president of Kenya rolled out the Big Four agenda in which attainment of Universal health coverage (UHC) for all by 2022 was prioritised. The goal of UHC is to provide access to quality health services to all people and communities without suffering financial hardship 32. Free maternity services were introduced in all public hospitals in June 2013, leading to a reduction in maternal and perinatal mortality as more babies were being birthed in hospitals 31. The country has an immunization coverage of over 80% which makes immunization clinics an alternative screening platform for those babies who are not born in health facilities 34. The Ministry of Health launched the National Plan for Ear and Hearing Care in August 2016, with one of its mandates being to initiate and develop early hearing detection and intervention services 35. This was done in pursuance of two World health Assembly resolutions which urges member states to establish national plans for ear and hearing care and to integrate strategies for ear and hearing care within the framework of their primary health care systems 36,37. Newborn hearing screening programs are yet to be established in the country, though a few private hospitals have been running infant hearing screening programs in immunization clinics. The objective of this study was to determine the prevalence of hearing loss among newborns and infants with a view to establishing early hearing detection and intervention programs in Kenya.

## Materials and methods

### Study setting and design

The study was conducted at the Kenyatta National Hospital (KNH) maternity ward, newborn unit (NBU) and newborn intensive care unit (NICU) in the department of reproductive health and at the Mbagathi subcounty hospital maternal child health clinic, in Nairobi, Kenya. This was a cross-sectional study and convenient sampling was used to enrol the study participants. Ethical approval to conduct this study was obtained from the Kenyatta National Hospital and University of Nairobi (KNH/UON) ethical committee.

### Study personnel

The study team comprised four registered community health nurses who were recruited as research assistants to facilitate data collection, a project manager, data manager and data clerk who were all working under the supervision of the principal investigators. The project manager's role ensured project activities were conducted as per the study protocols and contacted the parents and care givers for follow-up appointments. The data manager and clerk had the responsibility of collecting all the study questionnaires from the research assistants on a daily basis, ascertaining that all the fields in the questionnaires were filled and conducting data entry. Each research assistant was assigned a study code. The principal investigators conducted a three-day focused training for the research assistants on all the study protocols and procedures which included how to obtain informed consent, conduct hearing screening using the DPOAEs and AABR equipment. The diagnostic evaluation was conducted by an Audiologist.

### Test procedures

#### Collection of case history information

The case history information was obtained from the baby's file and from interviews with the mother. Consent to conduct the hearing screening was sought from the mother after an explanation about the purpose of the test and how it would be done was given. This was followed by a clinical examination on the baby. Babies with eternal auditory canal atresia in both ears were excluded from the study.

#### Hearing screening

**Figure 1:**
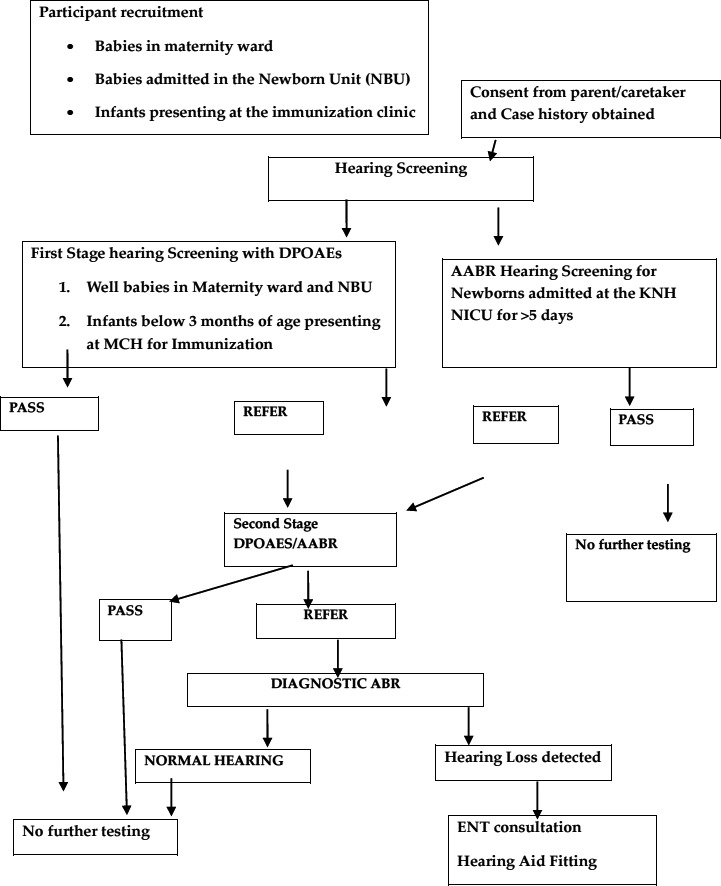
Study Flowchart

Hearing screening was conducted on all babies in the KNH maternity ward, newborn unit, neonatal intensive care unit and babies less than 3 months of age presenting at the MCH clinic for the immunization scheduled at 6 weeks and 10 weeks of birth (Figure 1). The well babies were discharged between 12-24 hrs after birth and those born through Caesarean section were discharged after 3 days. A case history was obtained followed by hearing screening with Distortion Product Oto-acoustic emissions (DPOAEs) and automated auditory brainstem response (AABR). Hearing screening was conducted closer to the time of hospital discharge on all days of the week except on Sunday's. DPOAE screening was conducted by presenting a click sound stimulus through a small probe placed in the ear canal. The hearing screen was considered an overall pass when at least three pass results were obtained out of the four frequencies tested between 2-5 kHz in each ear. For AABR screening, a click sound stimulus was presented to the ear through an insert earphone placed in the baby's ear canal. Surface electrodes were placed on the baby's head to record the response. The results for both the DPOAEs and the AABR were displayed either a “pass” or “refer”. For DPOAEs screening, primary tones were presented at levels of L1= 65dB and L2 = 55 dBSPL. A signal-to-noise ratio of 6 dB in three out of four frequencies tested between 2-5 KHz was required to qualify for a pass. The screening procedure was done at the bedside or in a quiet side room after the baby was feed. The screening results were recorded and an explanation of the results given to the mother. No further testing was done for babies whose screening results showed a pass. Those who referred were given a follow-up appointment within six weeks of hospital discharge. At the follow-up appointment, a twostep hearing screening with DPOAEs and AABR was conducted. Those who referred the second stage rescreening, were referred for diagnostic ABR at the KNH Otorhinolaryngology clinic. All babies admitted in NICU for more than 5 days underwent initial AABR hearing screening before discharge. No further testing was done for those who passed the AABR screen. Those who referred AABR were given an appointment for a rescreen within six weeks. Diagnostic ABR was done within 3 months for babies who referred the rescreen with no further testing performed for those with normal ABR results.

#### Confirmation of hearing loss

Diagnostic tone burst ABR was conducted by presenting the sound stimulus through an insert earphone placed in the baby's ear canal. Surface electrodes were placed on the baby's head to record the ABR response. In this study, hearing loss greater than 30 dBnHL whether bilateral or unilateral was considered as permanent congenital hearing loss. A referral was made to the ear, nose and throat specialists upon confirmation of hearing loss which was followed by auditory habilitation.

#### Data management and quality control

A data management and analysis schedule were developed to ensure proper handling of data from data collection to analysis. Questionnaires were consistently checked for completeness at the study site on a daily basis before submission to the data centre. Patient identity was anonymized to conform with confidentiality requirement. Quantitative data from the field questionnaires was entered into a computer database designed using MS-Access application. Regular data backup was done using external storage drives for data recovery in the event of data loss. In addition, approximately 500 records were randomly selected across all the entry records and double entered for comparison purposes. This assisted in checking on the quality of data entry. On completion of data entry, cleaning and validation was performed in order to achieve a clean dataset that was exported into a Statistical Package format (IBM SPSS version 25.0) for analysis. Data analysis was performed using IBM SPSS version 25.0 statistical software. Descriptive statistics such as frequencies and proportions were used to summarize all categorical variables. Pearson's Chi-square test was used to test for the difference in case referral across points of enrolment of the study participants. Threshold for statistical significance was set at p<0.05. Data collection tools were tested and validated prior to data collection. Questionnaires were checked daily for completeness and consistency before data entry. In order to verify the quality of data collected, the 25% of the babies were randomly selected for a repeat hearing screening and a counter check of the results.

## Results

A total of 10,104 babies were eligible for screening; of these 9,963 of the mothers consented to participate and assented for their babies to be screened. Those who were not screened for reasons of early discharge or death of baby were 1.4% (141/10,104) yielding a screening coverage of 98.6% (9,963/10,104). The results are presented in three sections: (1) Background characteristics (2) Screening results (3) Confirmation of hearing loss.

### Background characteristics

Table 1 presents the neonatal and maternal background characteristics. The highest number of enrolments came from maternity ward (82.2%), with 12.9% from the NBU and 4.9% from the Immunization Clinic. Of the 1287 new-borns admitted to the NBU, 56.6% were admitted to NICU. Of the 728 admitted to the NICU, 23.5% stayed for more than 5 days. Most of the deliveries reached full term (48.8%) or early term (31.1%), with 8.5% born preterm. There was a comparable male (50.9%) to female (49.1%) distribution among the infants. A majority of the infants (89.0%) had normal birth weight, with 8.7% having low birth weight and 0.4% with very low birth weight. Almost all infants (99.5%) were born in a health facility (91.8% born in KNH) with 0.5% being home deliveries.

**Table 1:** Neonatal and maternal background characteristics

Variables	N=9963	%
**Location code**		
Maternity Ward	8188	82.2
NBU	1287	12.9
Immunization Clinic	488	4.9
**Patient admitted to NICU from NBU**		
Yes	728	56.6
No	559	43.4
**Length of stay in NICI, in days (n=728)**		
> 5 days	171	23.5
< = 5 days	557	76.5
**Gestation age classification (in weeks)**		
Preterm (<34 weeks)	123	1.2
Late preterm (34 - 36 weeks)	727	7.3
Early term (37 - 38 weeks)	3097	31.1
Full term (39 - 40 weeks)	4859	48.8
Late term (41 - 42 weeks)	1063	10.7
Post term (>42 weeks)	94	0.9
**Gender of the baby**		
Male	5069	50.9
Female	4894	49.1
**Birth weight**		
Very low (<1500g)	43	0.4
Low (1501g - 2499g)	863	8.7
Normal (2500g - 4200g)	8864	89.0
Overweight (>4200g)	193	1.9
**Place of birth**		
Health facility	9913	99.5
Home	50	0.5
**Health facility (n=9913)**		
KNH	9103	91.8
Other	810	8.2

### Screening results

The number of babies referred at the initial screening is illustrated in Figure 1 and Table 2 which indicates an overall referral rate of 3.6% (356/9,963). The majority referred at the initial screening were from the Maternity unit, 88.2% (314/356), followed by the immunization clinic, 8.1% (29/356) and lowest from the New-born Unit, 3.6% (13/356). Thereafter, 258 of the 356 babies (72.4%) returned for follow-up rescreening, 233 from Maternity unit (65.4%), 16 from Immunization clinic (4.5%), and 9 from the New-born Unit (2.5%). A total 98 of the 356 babies (27.6%) were lost to follow-up, 81 from Maternity unit (22.8%), 13 from Immunization clinic (3.7%), and 4 from the New-born Unit (1.1%). There was a significant difference in proportion of case referrals across points of enrolment (p<0.001), the highest referral observed at immunization clinic (5.9%, 29/488), followed by maternity (3.8%, 314/8188), and the lowest at new-born unit (1.0%, 13/1287).

**Table 2:** Summary of initial screening results

Screening Outcomes	Maternity ward (n=8188)	Newborn Unit (n =1287)		Immunisation (n=488)	Total (n=9963)
		NBU	NICU		
PASS	7874	551	723	459	9607
REFER	314	8	5	29	356
TOTAL	8188	559	728	488	9963

### Confirmation of hearing loss

All 26 babies referred for diagnostic hearing testing returned and were confirmed to having hearing loss of 30dBnHL or greater (Table 3). A total of 21/26 (80.9%) infants had bilateral hearing loss and five 5/26 (9.1%) had unilateral hearing loss. Six babies (23%) had mild hearing loss (26-40 dBHL), eleven (42%) had moderate hearing loss (41-60 dBHL), six (23%) had severe hearing loss (61-80 dBHL) and 3(12%) had profound hearing loss (> 81 dBHL). The prevalence of confirmed diagnosis of hearing loss for all categories of hearing loss was 0.3% (95% CI: 0.2% - 0.4%).

**Table 3:** Summary of diagnostic results

Summary of Diagnostic Results			
Categories of Hearing Loss	Bilateral	Unilateral	n= 26 (%)
Mild 26-40 dBnHL	4	2	6 (23)
Moderate 41-60 dBnHL	9	2	11(42)
Severe 61-80 dBnHL	5	1	6 (23)
Profound >81dBn HL	3	0	3 (12)
**Total**	**21/26**	**5/26**	

## Discussion

The incidence of disabling hearing loss is increasing, with recent estimates indicating that almost half a billion people have such hearing impairment in 2018 8. Of these, about 7.5 million are children under the age of 5 years, with a large percentage in developing countries, particularly those in Sub-Saharan Africa 38. Globally hearing loss is now the 4th leading cause of years lived with disability. A majority of the morbidity associated with neonatal and childhood hearing loss is preventable, in large measure through universal newborn hearing screening programs.

This pilot study in Kenya is important in providing necessary information to help initiate universal newborn and infant hearing screening (UNHS) programs in the country. The study attained a screening coverage of 98%, exceeding JCIH recommended screening coverage of 95%. This screening coverage was comparable to that obtained in other developing countries, 98.7% (Nigeria) 13 and 95% (South) 27. There was maternal willingness to participate in the screening process which was achieved through educating mothers on the importance of the hearing screening in early detection of hearing loss and the effects hearing loss can have on a child's development of speech and language. None of the mothers declined to give consent for screening. The short hospital stay of between 12-24 hours for well babies contributed to some babies being discharged before the hearing screening was done. Babies delivered through caesarean section and those admitted in the newborn unit had minimum hospital stay of three days which made it possible to have all of them screened before discharge. The nursing staff were sensitized on the need to have all babies screened before discharge with a view to optimising screening coverage through their cooperation. All mothers whose babies underwent hearing screening were given an informational booklet which included the hearing screening results. A referral letter was issued to those who required follow-up screening with information that there would be no fees charged for follow-up outpatient visits. The higher percentage of hospital births (99.5%) in this study compared to home births (0.5%) can be attributed to the free maternity services provided in public hospitals.

The overall referral rate of 3.6% obtained in this study was within JCIH recommendations of no more than 4% for a UNHS program. The referral rate for infants less than 3 months of age attending the immunization clinic was 5.9% which was higher than that of well babies in the maternity ward (3.8%) and NBU (1.0%). Higher referral rates have been found for OAE only screening protocols, 11% (South Africa) and lower referral rates where two step screening protocols are used, 3.5% (Nigeria) 2.2% (India), 2.0% (Hong Kong), 1.33% (Saudi Arabia). 13,29,39,40. Higher referral rates are expected where babies have a short duration of hospital stay due to presence of vernix caseosa in the ear canal, middle ear fluid and screening in a noisy environment. The lower referral rates obtained in this study could partly be attributed to the low ambient noise levels which were achieved by conducting screening after the routine ward activities and babies feeding times. Screening was done at higher frequencies of 2-5Khz which has been found to be associated with lower referral rates as its less likely to be affected by vernix and middle ear fluid 41.

Tracking for follow-up rescreening and diagnostic testing was done through mobile telephone calls to the parents or caregivers. The return rate for follow-up rescreening was 72.4% which is below the recommended rate of more than 95%. 22, 23. Poor follow-up return rates have been reported as a challenge for hearing screening programmes. 27,29,42,43,44. The major contributing factors to loss of follow-up in this study was parental reluctance for further evaluation and an inability to track those who gave incorrect telephone contacts or numbers that belonged to relatives, friends or neighbours. Some mothers had travelled back to their rural homes and were unable to meet travel costs for follow-up appointments. The return rate for diagnostic hearing evaluation was 100% (26/26) which was as a result of enhanced parental education and counselling on the importance of the return visit, aligning the appointment with other neonatal follow-up clinics and providing mothers a choice with a selection of choice of return dates to avoid missed appointments. The prevalence of hearing loss in this study was found to 3/1000 (Table 3).

It is critical to have a continuum care from screening, to validation of the screening results though diagnostic audiology testing, to effective interventions such as hearing aids, cochlear implants, sign language instruction, to addressing the stigma of hearing impairment in the society. This continuum of care also requires comprehensive infrastructure, including tracking capability through efficient data systems and trained personnel, adequate numbers of trained screeners as well as audiologists, and public funding through the Ministry of Health. Full time staff are essential for the success of such a UNHS program. The staff at the hospital received two weeks of training prior to commencement of the screening program. Another factor which is important to implementing UNHS successfully is the support of the medical community, particularly primary care physicians 45. The medical school curriculum should include the huge impact of neonatal hearing impairment on long term development of children into adulthood, and the economic cost over a lifespan. The cost effectiveness of newborn screening should be included, along with a description of newborn screening tests, such as OAE and AABR.

Screening for hearing impairment can be coordinated with other universal health interventions, such as immunization programs, to make them more cost-effective. The Ministry of Health provides a mother and child health (MCH) handbook which is used to record a child's health record from newborn up to age 5 years. We recommend the inclusion of hearing screening into the MCH handbook so that this can be conducted during the immunization clinic visit.

One of the large benefits of an effective screening program is the ability to reassure the vast majority of parents that their child has normal hearing. Sustaining a successful UNHS program requires both expertise and passion. Selecting a champion(s) who have both charisma as well as strong leadership qualities is critical. This may be a professional such as an audiologist or otolaryngologist, a parent, a child, or a public figure who has hearing impairment.

## Conclusion

UNHS is an effective program for early identification and intervention for hearing impairment. A well-coordinated multidisciplinary approach involving health professionals such as Audiologists, nurses, paediatricians, medical specialists, government policy makers in health and education and parents is critical to making this program effective. The sustainability and effectiveness of the UNHS program will depend on government goodwill and policies demonstrated through the allocation of funds for human resource, equipment, hearing devices and training of hearing health care workers.

## Figures and Tables

**Figure 2:**
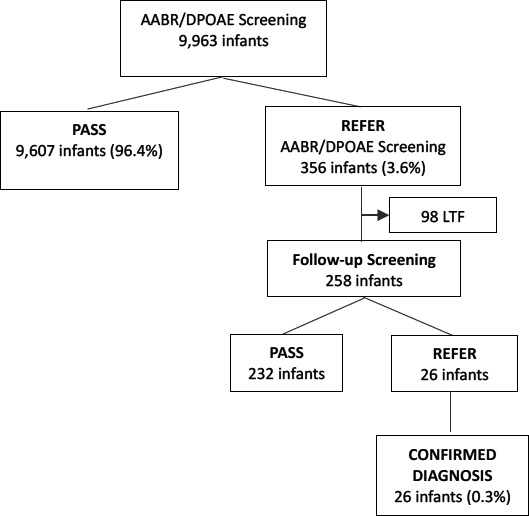
Flow chart on the diagnosis of hearing loss
